# Nanopore sensing at ultra-low concentrations using single-molecule dielectrophoretic trapping

**DOI:** 10.1038/ncomms10217

**Published:** 2016-01-06

**Authors:** Kevin J. Freedman, Lauren M. Otto, Aleksandar P. Ivanov, Avijit Barik, Sang-Hyun Oh, Joshua B. Edel

**Affiliations:** 1 Department of Chemistry, Imperial College London, South Kensington, London SW7 2AZ, UK; 2 Department of Electrical and Computer Engineering, University of Minnesota, Minneapolis, Minnesota 55455, USA; 3 Department of Biomedical Engineering, University of Minnesota, Minneapolis, Minnesota 55455, USA

## Abstract

Single-molecule techniques are being developed with the exciting prospect of revolutionizing the healthcare industry by generating vast amounts of genetic and proteomic data. One exceptionally promising route is in the use of nanopore sensors. However, a well-known complexity is that detection and capture is predominantly diffusion limited. This problem is compounded when taking into account the capture volume of a nanopore, typically 10^8^–10^10^ times smaller than the sample volume. To rectify this disproportionate ratio, we demonstrate a simple, yet powerful, method based on coupling single-molecule dielectrophoretic trapping to nanopore sensing. We show that DNA can be captured from a controllable, but typically much larger, volume and concentrated at the tip of a metallic nanopore. This enables the detection of single molecules at concentrations as low as 5 fM, which is approximately a 10^3^ reduction in the limit of detection compared with existing methods, while still maintaining efficient throughput.

## Abstract (web summary)

Nanopore sensors have shown tremendous potential for biomolecule sensing, though the diffusion-controlled capture can limit the speed of analysis. Here, the authors report a dielectrophoretic method to concentrate DNA near the tip of a nanopore, reducing the limit of detection by three orders of magnitude.

## Graphical abstract

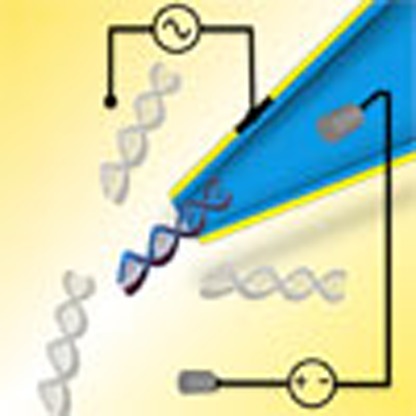

One of the greatest challenges facing physical and biological scientists is the accurate detection and identification of single molecules. The drive to performing such experiments is a result of experimental observations typically being an ‘ensemble average' whereas a single-molecule measurement enables the possibility of detecting kinetics and dynamics from individual molecules in real time. Since the mid 1990s, nanopores have been a rapidly growing technology for use in applications such as single-molecule DNA sequencing^1^,^2^,^3^, protein detection^3^,^4^,^5^,^6^ and the study of RNA-drug targets^7^ largely owing to the method being label-free while still maintaining a high temporal resolution. As a consequence, nanopore sensors have the potential to revolutionize the healthcare industry with numerous clinical applications already in the pipeline. Although promising, nanopore sensing has several drawbacks with one of the most problematic being the lack of efficiency when trying to detect individual molecules from the bulk solution. In fact, this problem extends further and is fundamental to many surface-based biosensors^8^. This stems from the fact that the dominant mechanism of capture and detection (for example, the process of DNA translocating through the pore) is diffusion-limited resulting in only a small fraction of the total sample volume being accessed. For example, a 10 μl solution at a concentration of 1 nM will have on average 34 molecules in the capture volume assuming a capture radius of 3 μm. This is roughly 10^8^ times lower than the actual number of molecules in the solution. At sub-picomolar concentrations, the average number of molecules inside the capture volume would be well below 0.03 leading to long measurement timescales (>1 h). It is clear that new strategies are needed to enable ‘rare event' and ‘needle-in-a-haystack' detection experiments.

Importantly, molecular transport through nanopores has been studied extensively^9^,^10^,^11^, however, there have been much fewer documented techniques which have demonstrated capture rate enhancement. In 2010, the limit of detection was reduced from several hundred picomolar to 3.8 pM by utilizing high salt gradients to manipulate the voltage drop outside the nanopore^12^. Using this technique, an event rate of ∼60 molecules per minute was achieved. More recently, a subclass of nanopores called nanopipettes was shown to offer similar levels of sensitivity by loading DNA inside the pipette and applying voltage pulses to improve the efficiency of delivery from the nanopore^13^. Protein pores also offer unique biological methods of increasing the translocation rate including modifying the internal charge of the pore^14^,^15^. Both studies made use of the electrophoretic properties of DNA to enhance the capture rate, while others have attempted to use pressure gradients to add a level of control to the translocation process^16^. However, all these methods have limited ability to concentrate and perform high-throughput detection of ultra-dilute samples. Although these studies have focused on DNA, it should be noted that the goal to increase capture rate has far-reaching applications related to rare event detection and is not exclusively directed towards DNA sequencing; especially if DNA translocations are successfully slowed down such that the inter-event time is not a limiting factor.

In this manuscript, we developed a novel method to improve the detection efficiency by 1,000-fold by incorporating a dielectrophoretic (DEP) trap at the nanopore opening. The DEP trap not only provides on-demand control of the capture volume (that is, the volume in which molecules become drawn towards the pore) but can also significantly increase the number of molecules being detected per unit time even at concentrations of a few femtomolar. The reported technique pushes the envelope of high-sensitivity and amplification-free single-molecule detection and paves the way for high-speed and high-throughput detection of ultra-dilute samples and rare events.

## Results

### Nanopipettes incorporating dielectrophoretic traps

**Figure 1:**
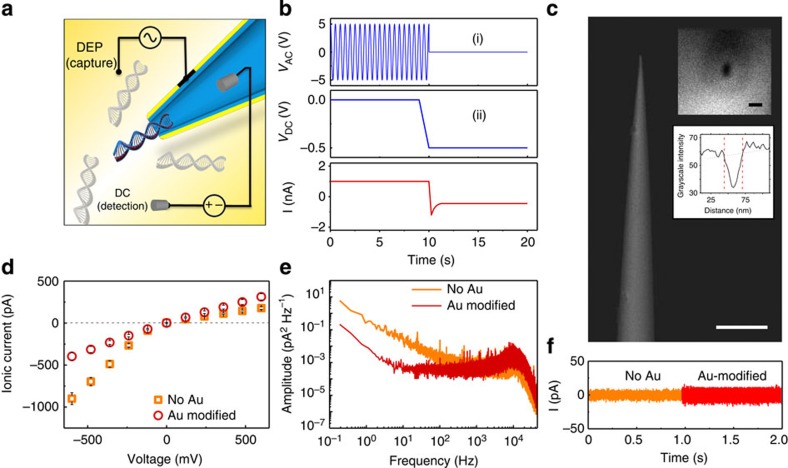
Experimental setup and characterization of gold-coated nanopipettes. (**a**) Schematic of DNA being threaded through the tip of a gold-coated nanopipette. (**b**) Schematic of the voltage protocol used for DEP pre-concentration phase via AC voltage (i) and subsequent nanopore translocations via DC voltage (ii). All three channels (*V*_AC_, *V*_DC_, *I*) are simultaneously recorded. (**c**) SEM of a gold-coated nanopipette; scale bar, 5 μm (insets: SEM and intensity line plot of the tip visualized parallel to the barrel; scale bar, 50 nm). (**d**) Current–voltage curves for glass nanopipettes before and after gold coating. Gold coating thickness was ∼5 nm. (**e**) Power spectral density of pipettes under a negative 500 mV voltage bias. (**f**) Baseline-subtracted time traces of the pipettes before and after gold coating at a negative 500 mV voltage bias and 1 mM KCl.

Typically, nanopore experiments use a constant DC bias to prompt the translocation of an analyte molecule. In this study, an AC voltage (10–20 V and 0.5–4 MHz) is applied to the metallized layer surrounding the nanopipette followed by a DC voltage which translocates the molecules, Fig. 1a. The AC and DC voltages are applied to the system using two Au electrodes and two Ag/AgCl electrodes, respectively (Fig. 1a,b). Metallic tips and apertures, used for DEP experiments and biosensing^17^, offers several key advantages over planar electrodes which include (i) a three-dimensional trapping volume, (ii) being able to control the electrode gap distance and therefore the field gradient forces and (iii) added enhancement due to the sharpness of the metallic tip^18^.

Nanopipettes are glass or quartz capillaries that are heated and pulled until a nanometer-sized aperture is formed at the tip. When biological molecules are passed through the nanopore, the exclusion of ions causes a decrease in bulk ion flow thereby allowing single molecules to be detected. Although it is in principle possible to measure the ionic current in the presence of a superimposed AC field to enable molecular trapping, calibration of the competing DEP and electrophoretic forces are far from trivial. For simplicity, experiments were split into a DEP (capture) phase (Fig. 1b, (i)) and an electro-kinetic translocation (detection) phase (Fig. 1b, (ii)). To adapt the nanopipettes (25 nm diameter) for use in dielectrophoretic trapping, a thin 5 nm layer of Au was deposited on the barrel (Fig. 1c) and were characterized by filling with 1 mM KCl and measuring the ionic current when a DC voltage bias is applied between the Ag/AgCl electrodes (Fig. 1a). Further details of the experimental setup and nanopipette positioning are available in Supplementary Note 1 and Supplementary Figs 1 and 2. Using uncoated quartz nanopipettes, the current–voltage (*I*–*V*) curve showed rectification (that is, unequal conduction depending on voltage polarity) consistent with that expected by negatively charged conical glass nanopores^19^,^20^. On coating with gold, the rectification ratio is reduced but is still present (|*I*_−600 mV_/*I*_600 mV_|=1.3) compared with the bare pipette (|*I*_−600 mV_/*I*_600 mV_|=4.8; Fig. 1d), indicating a reduction in the surface charge on the pipette^13^,^21^,^22^,^23^,^24^. The noise characteristics after gold deposition showed more than an order of magnitude reduction in low frequency 1/*f* noise typically associated with conductance fluctuations (flicker noise) and a mild increase in higher frequency noise attributed to capacitance (representative plots of >50 nanopipettes shown in Fig. 1e,f)^25^. Importantly, while measuring ionic current, relatively small changes in the noise demonstrated that metallized nanopipettes are suitable for the envisioned application of DEP-enhanced DNA detection.

### Modelling of dielectrophoretic fields

Incorporating a DEP trap to the nanopore was achieved by adding gold to the area surrounding the nanopore (that is, the nanopore entrance), as demonstrated by the change in ionic conductance. With the gold electrode in proximity to the pore, high gradient forces can be generated. However, theoretical approaches are needed to find the distribution of the electric fields around the tip of the nanopipette. The counterion fluctuation (CIF) model is proposed as the best possible method for describing the true nature of the polarizability of DNA, which is based on the redistribution of the counterions from the solution which surround the molecule near its charged sites, see Supplementary Note 2 and Supplementary Figs 3–5. The polarizability of the 10 kbp DNA used in experiments was calculated to be 1.59 × 10^−30^ F m^2^ and was determined by normalizing the subunit polarization, α_s_, to the entire length of the DNA (see Supplementary Information for further details). The polarizability of the DNA from the modified CIF model was multiplied with the electric field intensity gradient extracted from finite-element method (FEM) models to determine the force on the DNA molecules.

**Figure 2:**
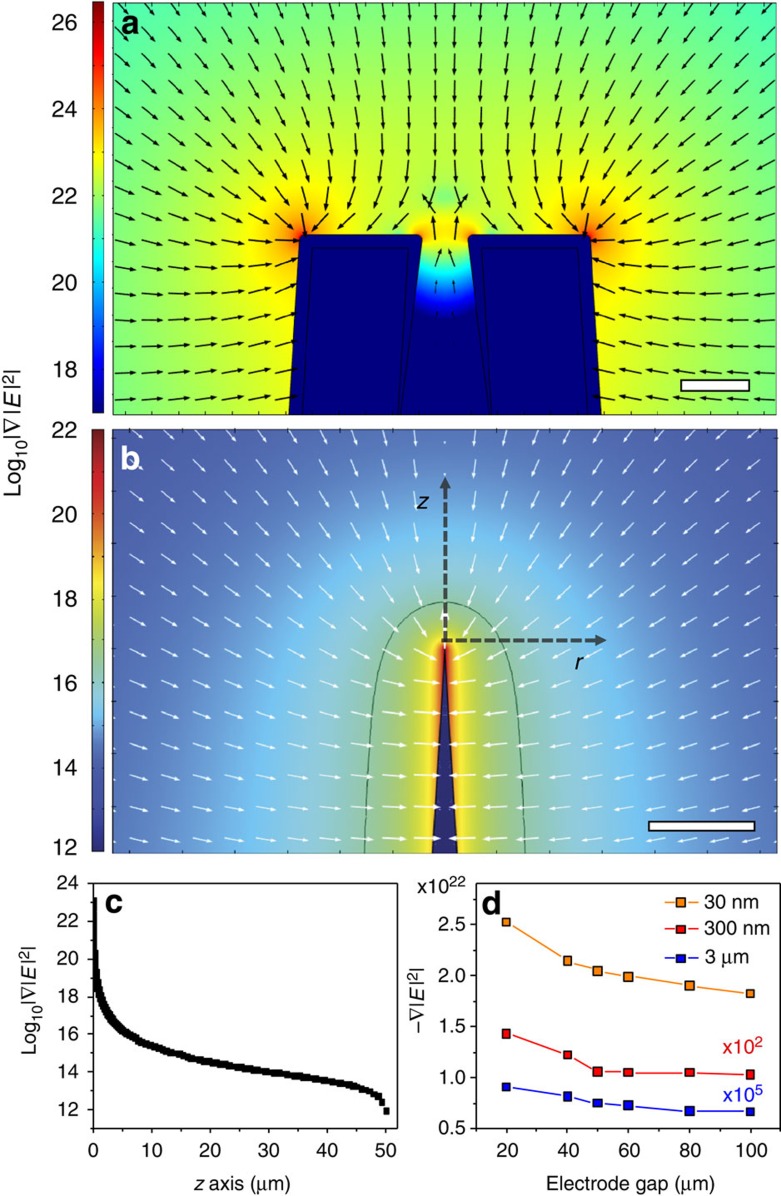
Finite-element method modelling of DEP with the nanopipette. Two-dimensional-axisymmetric electrostatic modelling of a 50 μm electrode gap between the nanopipette tip and a conductive flat electrode was simulated with a 10 V DC signal applied. Plots of the magnitude of the field intensity gradient, which is proportional to the force on a particle, are shown for regions (**a**) near the end of the pipette (scale bar, 25 nm) and (**b**) the DEP trapping volume and surrounding area (scale bar, 10 μm). The logarithmically scaled arrows show the direction of the force. The black contour line in **b** is along |∇|*E*|^2^|=10^16.4^ V^2^ m^−3^, which corresponds approximately to the threshold field intensity gradient for trapping 10 kbp DNA against Brownian motion. (**c**) The magnitude of the field intensity gradient along the *z* axis (black dashed arrow) in **b**. (**d**) The strength of |∇|*E*|^2^| was also tracked at different distances from the tip of the nanopipette along the *z* axis for electrode gaps ranging from 20 to 100 μm with a 10 V DC applied signal. The field intensity gradient strength decreases logarithmically with increasing gap size.

FEM modelling was performed using COMSOL Multiphysics 4.3a to determine the strength of the electric field intensity gradient, |∇|*E*|^2^|. Plots of log_10_|∇|*E*|^2^| surrounding the nanopipette were extracted from the model to show the strength of the electric field intensity gradient, and therefore the force, near the nanopipette (Fig. 2a) and several micrometers from the nanopipette (Fig. 2b). Critical to DEP trapping, it was discovered that DEP forces are strongest at the edges of the nanopipette tip and therefore DNA will be drawn directly to the nanopore. The threshold force (that is, the force required to overcome Brownian motion: 9.92 fN) extends ∼4–5 μm from the nanopipette tip in our models (marked with a black contour line at the threshold |∇|*E*|^2^|=10^16.4^ V^2^ m^−3^). It should also be noted that the threshold force and therefore the trapping volume is adjustable based on the parameters of the AC voltage applied to the pipette.

The DEP force acting on the particles under fixed conditions (10 V, 1 MHz) is shown by plotting the log_10_|∇|*E*|^2^| (Fig. 2c) along the *z* axis extending out from the nanopipette tip toward the flat electrode which is 50 μm from the nanopipette tip. One of the key parameters that the simulations probed was the distance between the nanopipette tip and the planar electrode, *d*_gap_, which was experimentally set using a micromanipulator. In Fig. 2d, the dependence of −∇|*E*|^2^ along the *z* axis is shown for both changing distances from the nanpopipette tip (30 nm, 300 nm and 3 μm), as well as changing electrode gap distances (*d*_gap_=20–100 μm). Function fitting shows a logarithmically decreasing trend in the force as the electrode gap distance increases, which matches our expectations based on previous literature^26^.

## Discussion

### Single-molecule fluorescence imaging of dielectrophoretic traps

**Figure 3:**
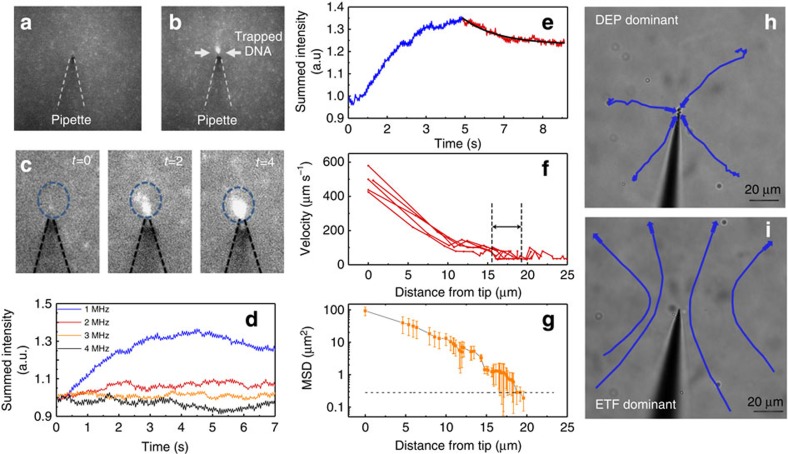
Fluorescence images and analysis of DNA trapped at the nanopipette tip. (**a**) YOYO-labelled 10 kbp DNA sample with the gold-coated nanopipette positioned 50 μm above the surface of the planar counter electrode without applied AC voltage. (**b**) YOYO-labelled 10 kbp DNA sample with an AC voltage (*V*_pp_=20 V, *f*_AC_=1 MHz) being applied between the gold layer on the nanopipette and the counter electrode. (**c**) Images showing the progression of DNA trapping which occurs at the tip of the nanopipette. (**d**) Fluorescent intensity plotted over time for video recordings conducted at *V*_pp_=20 V and *f*_AC_=1, 2, 3, 4 MHz. (**e**) Fitting the reduction of fluorescent intensity to an exponential (*τ*_decay_=1.54 s) for the 1 MHz condition. (**f**) DNA velocity profiles for five 10 kbp DNA molecules being trapped using the following conditions: *V*_pp_=12 V, *f*_AC_=1 MHz. (**g**) Average mean squared displacement (MSD) of 10 kbp DNA as they transition into the trapping volume. Black dotted line: MSD expected for diffusion alone. (**h**,**i**) Trajectories for 1 μm polystyrene beads showing DEP-dominant (*f*_AC_=1 MHz, *V*_pp_=12 V, *d*_gap_=50 μm) conditions and ETF-dominant conditions (*f*_AC_=1 MHz, *V*_pp_=20 V, *d*_gap_=200 μm).

Quantification of the DEP trapping kinetics was accomplished using YOYO-1 labelled 10 kbp DNA in conjunction with fluorescence microscopy. The key parameters which were controlled include the electrode gap distance (*d*_gap_), the AC frequency (*f*_AC_) and the AC peak-to-peak voltage (*V*_pp_). Qualitatively, once the AC field was turned on, the DNA would localize and surround the region of the pipette with the sharpest geometric features (that is, the tip of the nanopipette, Fig. 3a,b), as expected from simulations^17^. As the trapping field was kept on for longer time periods, the fluorescent region around the tip would grow in size owing to the accumulation of labelled DNA (Fig. 3c). At *d*_gap_=50 μm and *V*_pp_=20 V, various frequencies were applied to the two gold electrodes. Using the CIF model, the frequency dependence is owing to the relaxation time constant of the ions surrounding the DNA; specifically the time duration for charges to relax after being perturbed by an electric field. For a 12 kbp DNA molecule and a similar dielectric decrement, the relaxation frequency was ∼2 MHz (ref. 27), which explains the sudden loss of DEP trapping efficiency at the higher AC frequencies (2, 3 and 4 MHz). Indeed the highest fluorescence intensity was obtained for the 1 MHz condition, which maximizes at about 4.5 s after applying the AC field. Interestingly, a decrease in the intensity past this point is observed which is likely due to the DNA closest to the tip (trapped within the focal plane for the most time) being photobleached (exponential decay in intensity shown in Fig. 3e)^28^,^29^.

To characterize the effects of DEP acting on the DNA, the spatial position of the DNA relative to the tip was tracked using image processing. As the DNA diffuses close to the tip (16–19 μm), the velocity of the DNA increased as the molecules enter a region of AC field-induced motion (DNA trajectories plotted in Fig. 3f). The mean squared displacement (MSD) of DNA was calculated using the two-dimensional (2D) diffusion equation (<*x*^2^>=4Dt), as the optical images represent a projection of the three-dimensional molecular movement into a 2D image. The MSD was averaged over two frames (15 ms per frame) and plotted against distance away from the nanopipette tip (Fig. 3g; D_10 kbp DNA_=1.05 × 10^−8^ cm^2^ s^−1^; ref. 30). The point where DNA crosses over from a diffusion-limited regime to an AC field-enhanced regime occurred between 16 and 19 μm from the tip (marked by arrows in Fig. 3f).

Interestingly, DEP is not the only force experienced by DNA. Electrothermal flow (ETF), stemming from temperature inhomogeneity in the fluid medium surrounding the pipette, became apparent at elevated voltages, see Supplementary Notes 3 and 4 and Supplementary Fig. 6. To demonstrate the distinct forces observed for both DEP and ETF, polystyrene beads were tracked at the conditions used for DNA trapping (*V*_pp_=12 V, *f*_AC_=1 MHz, *d*_gap_=50 μm) as well as at higher voltages and a larger electrode gap distance (Fig. 3h,i; *V*_pp_=20 V, *f*_AC_=1 MHz, *d*_gap_=200 μm). As DNA trapping experiments were performed at frequencies of 1 MHz and higher, we do not expect electro-osmotic flow to play a significant role. Although Fig. 3h,i show rather distinct trajectories, in reality, both DEP and ETF exist around the nanopipette and play a role in the trapping of DNA. For example, instead of DNA diffusing into the DEP-trapping volume, the ETF plays a role in enhancing the capture efficiency by delivering DNA into the trapping volume. An approximation of the trapping volume size for the above conditions is supplied in the Supplementary Note 5 and Supplementary Fig. 7, however, it should be kept in mind that the trapping volume is controllable depending on the applied AC potential. Therefore, the capture rate of DNA can be actively controlled by varying the DEP parameters.

### Single-molecule translocations and capture rate enhancement

**Figure 4:**
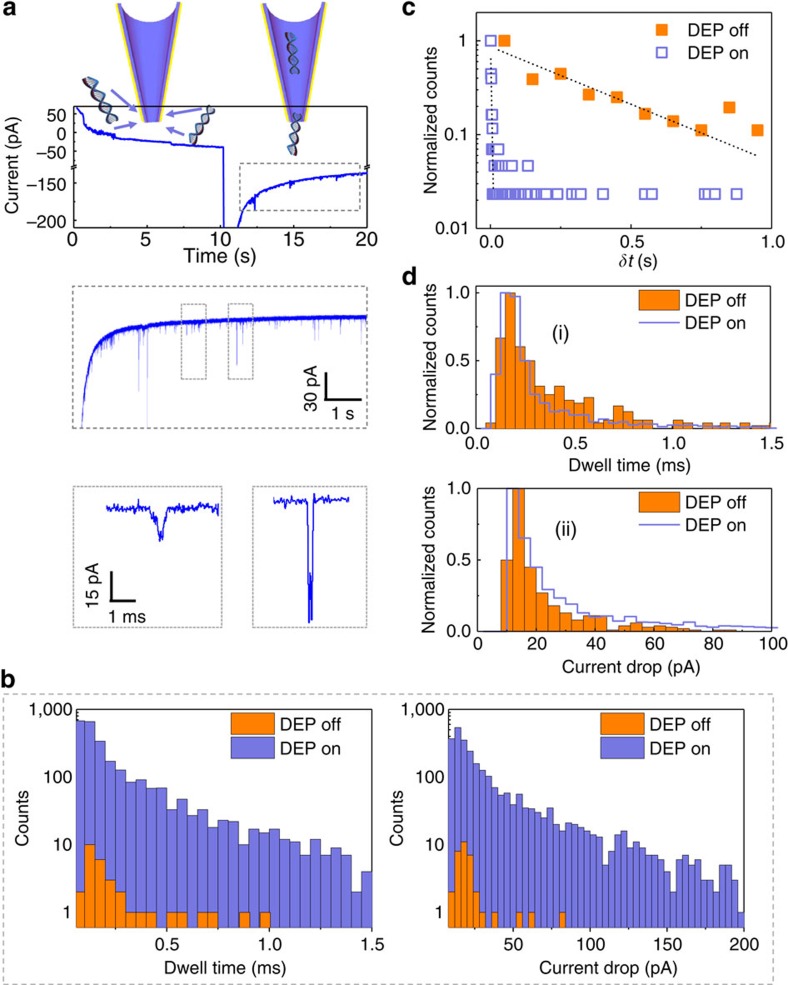
Single-molecule detection and analysis with and without DEP. (**a**) Current traces of a typical DEP pre-concentration/recording cycle with various time scaling. The first 10 s is the pre-concentration phase and the later 10 s is the translocation/detection phase. The lower panel shows typical events representing a single DNA molecule translocation (∼96% of events), as well as a DNA aggregate translocating the pore (∼4% of events; see Supplementary Note 7 and Supplementary Figs 13 and 14). (**b**) Time-normalized histogram of the dwell time and current drop comparing translocations with and without DEP pre-concentration. (**c**) Normalized histogram of the inter-event time (*δt*) with and without DEP pre-concentration. (**d**) Count-normalized distributions with and without DEP pre-concentration for the dwell time (i) and current drop (ii). All DEP data were obtained at *V*_pp_=12 V, *f*_AC_=1 MHz and *d*_gap_=50 μm.

The DEP phase of the experiment not only results in efficient molecule capture close to the pore entrance, but also enables the subsequent translocation. Using a DNA (10 kbp) concentration of 500 pM, DEP trapping and subsequent DNA translocations were performed. The dwell time of the DNA was measured as the full width half maximum of the ionic current signature for each translocation event. For reference, single-molecule translocations without DEP trapping showed consistent ionic current blockade amplitudes, dwell times and charge distributions to what is expected for conventional nanopore experiments (see Supplementary Note 6 and Supplementary Figs 8 and 9). These values were then plotted as a histogram along with the data obtained without DEP trapping (Fig. 4b). The data were time-normalized (recording time=180 s) and plotted on a log scale to show both populations (linearly scaled plots are shown in Supplementary Fig. 10. Typical enhancement factors (EF=number of events with DEP/number of events without DEP) range between 80 and 100 for the conditions tested (DEP duration=10 s, *V*_pp_=12 V, *f*_AC_=1 MHz and *d*_gap_=50 μm). EF values (calculated using the same DNA concentration) differ from our previously mentioned definition of detection efficiency which strictly refers to the ability to detect reduced concentrations of DNA (that is, the limit of detection). Using one particular experiment with EF≈80, with and without DEP, the number of events were 2,715 and 34, respectively. Importantly, translocation characteristics are not affected by DEP trapping as shown by a count-normalized histogram (Fig. 4d). Similarly, the maximum current drop obtained for each event was plotted as a log-scale histogram (time-normalized) and a linearly scaled histogram (count-normalized; Fig. 4b,d). Importantly, pipette to pipette reproducibility was good, see Supplementary Fig. 11.

The inter-event time (*δt*) was extracted from the data by taking the difference between the start times of two consecutive events. The *δt* parameter has an exponential distribution which can be fitted by a linear curve on a log-scaled axis (as shown in Fig. 4d). Importantly, the *δt* values during experiments where DEP trapping was used show a marked decrease. This is expected as there are more events per unit time due to the pre-concentration effects of DEP. Alternatively, a cumulative histogram can be used to show the percentage of events which occur below a certain inter-event time (Supplementary Fig. 12). Comparing between experiments with and without DEP trapping, it was observed that 95% of inter-event times were below 8,750 ms when trapping was not used while the same number of events occurred below 400 ms with trapping forces being used to pre-concentrate the DNA.

**Figure 5:**
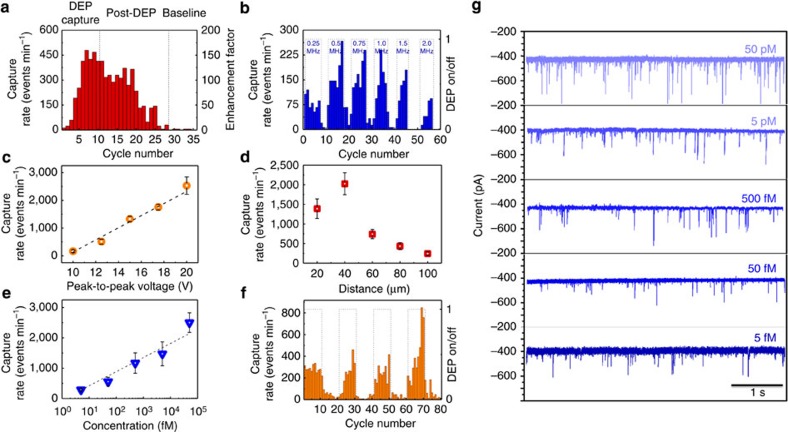
Analysis of enhanced capture rates. (**a**) Capture rate per recording cycle where each recording cycle was 10 s in duration. The first 10 cycles were performed with 10 s of DEP pre-concentration, whereas the last 25 cycles were proceeded by 10 s of no trapping forces. (**b**) Capture rate per recording cycle where DEP pre-concentration was turned on/off. The DEP trapping frequency was also increased with each subsequent DEP pre-concentration phase (*V*_pp_=12 V). (**c**) Capture rate as a function of the peak-to-peak voltage used for the DEP trapping (*f*_AC_=1 MHz). (**d**) Capture rate as a function of the nanopipette tip-counter electrode gap distance (*V*_pp_=12 V, *f*_AC_=1 MHz). (**e**) Capture rate as a function of the 10 kbp DNA concentration. (**f**) Capture rate per recording cycle for a 5 fM DNA sample where DEP pre-concentration was turned on and off for four consecutive cycles (*V*_pp_=20 V, *f*_AC_=1 MHz and *d*_gap_=20 μm). (**g**) Current traces obtained using DEP as a pre-concentration step for five different concentrations (50 pM, 5 pM, 500 fM, 50 fM and 5 fM). A bias of |*ΔV*|=500 mV was applied across the nanopore for all the experiments.

Further than simply increasing the capture rate before DNA translocations, multiple DEP cycles are shown to have a compounded effect on the capture rate of DNA. Typically the first cycle of trapping/translocation (1 cycle=10 s trapping+10 s translocations) yielded an increase in capture rate followed by further enhancement with each subsequent cycle (cycles with only 5 s of trapping were also tested; see Supplementary Note 8 and Supplementary Fig. 15). The trend shows an exponential increase in capture rate as a function of cycle number (Fig. 5a) up until equilibrium is reached, see also Supplementary Note 9. If it is assumed that each cycle is modelled as a rate balance (*R*_in_−*R*_out_=*R*_acc_) where the input of mass (that is, DNA) is governed by DEP and the output of mass is governed by the electrophoretic transport of DNA through the pipette, the increase in capture rate across cycles, *R*_acc_, is justified as being the result of an imbalance between these two rates. Losses due to diffusion are also present and make up a portion of the *R*_out_ term of the equation. The accumulation of DNA at the tip therefore seems to be a key contributor to the enhancement observed in our experiments.

By reducing the peak-to-peak voltage used for DEP trapping (20 to 12 V), the timescale for DNA accumulation, and post-DEP enhancement, was effectively reduced so that within two cycles, the capture rate fell to near-baseline values. The ability to reduce the post-DEP enhancement signifies that the DNA accumulation term was reduced and most of the DNA which was loosely bound to the pipette surface (that is, capable of desorbing) was removed by translocations. It is likely that the functionality of the nanopipette can be tuned to minimize surface adsorption to increase the collection efficiency. Cycles where DNA pre-concentration was used can clearly be identified by plotting the capture rate across cycles (Fig. 5b) where various AC frequencies were used in combination with a DC voltage of |*ΔV*|=500 mV.

The *V*_pp_ parameter used for DEP trapping is critical to the enhancement of DNA sensing as it determines the relative strengths of both DEP as well as ETF. As *V*_pp_ was increased from 10 to 20 V, a linear increase in the capture rate was observed. Surprisingly, while *V*_pp_ was only doubled at the extreme ends of the values tested (10 and 20 V), a 10-fold increase in the capture rate was discovered. Due to the presence of ETF, DNA is not delivered into the DEP capture volume by simple diffusion mechanisms but rather delivered at an enhanced rate via ETF. Furthermore, as ETF scales with voltage to the fourth power (*V*^4^; ref. 31), the strong dependence with *V*_pp_ is reasonable and provides further evidence for the existence of ETF in the nanopipette system^32^. In analysing the data presented in Fig. 5c–e, 10 consecutive DEP pre-concentrating cycles were used and each data point represents the average of the last five cycles.

To establish how the capture rate depends on the electrode gap distance, the pipette tip location was changed in 20 μm steps and DEP/translocation cycles were recorded for each position. As the gap between the two gold electrodes became smaller, the capture rate increased for gap distances between 100 and 40 μm (Fig. 5d), as expected from the FEM models (Fig. 2d). Interestingly, at 20 μm, the capture rate was reduced slightly. On the basis of the trapping volume data obtained using YOYO-1-labelled DNA described earlier, 20 μm is on the same scale as the distance away from the tip where DNA begins to respond to DEP forces. It is likely that the decrease in capture rate was due to the trapping volume (or ETF) becoming geometrically confined by the surface of the planar electrode.

High sensitivity detection in concentrations as low as 5 fM was achieved using a tip-to-surface gap distance of 20 μm and optimized DEP trapping conditions (*V*_pp_=20 V and 1 MHz). The capture rate logarithmically decreases as a function of bulk concentration (Fig. 5e). Although sub-picomolar DNA concentrations are typically not capable of being sensed using nanopores, DEP pre-concentration allowed the full range of fM concentrations to be detected (Fig. 5f,g, Table 1). Previous studies which used salt gradients, and later using controlled DNA delivery methods, showed the ability to enhance the capture rate of DNA down to ∼3 pM (refs 12, 13). Both of the previous methods rely on the electrophoretic properties of DNA to enhance the capture radius and the local concentration, respectively. The DEP-based method, which uses the polarizability of DNA, is shown here to be a much more powerful method for enhancing the capture rate of DNA. At 5 fM DNA, a capture rate of 315±147 events per minute is achieved (Fig. 5f). Before the experiments, a DC bias was applied to the same pipette for 2 min with a total of four events being detected. To obtain accurate event statistics, an arbitrary threshold of 1,000 events was deemed reasonable. If true, the recording time for the 5 fM sample would require a duration of 8.3 h. Using DEP, the recording time is 3.2 min.

We have demonstrated the use of metallized nanopipettes, and more generally nanopores, for DEP trapping and DNA pre-concentration. The foreseeable benefits of a hybrid DEP-nanopore device on genetic analyses are enormous owing to the need for ultra-sensitive methods that can analyse low concentrations of genomic DNA with high throughput. This report has demonstrated the ability to sense DNA at a concentration as low as 5 fM at an event rate of 315 events per minute. The capture rate enhancement is shown to stem from a larger DEP trapping volume in combination with enhanced delivery into the trapping volume by electrothermal flow. Importantly, the trapping volume size is a controlled and easily manipulated parameter which allows the capture rate to be changed on command. No longer is nanopore sensing dictated by the diffusion and electrophoretic properties of the analytes, but by their polarizability and the controlled AC voltage applied. Future work will be directed towards combining AC and DC voltages in an effort to apply two independent forces to the translocating DNA. Achieving this would enable the slowing down of the molecule providing access to novel sub-molecular information. The proposed DEP-based method of pre-concentrating an analyte using a nanopipette could also be extended to other analytes (proteins, RNA) as well as other spectroscopies including SERS where concentration is a critical parameter^33^,^34^,^35^,^36^. Most importantly, this device can bridge a major technological gap in science where currently there are few rare event detection strategies. DEP electrodes can be integrated into other nanopore systems such as solid-state pores using an additional photolithography step to deposit gold or any other conductive material. Protein pores either suspended in a polymer membrane or integrated into a silicon-based membrane^37^ could also benefit from DEP preconcentration. Finally, this platform opens up the door to single-cell experimentation whereby the sharp nanopipette tip can be used to efficiently extract low copy numbers of nucleic acids.

## Methods

### DNA labelling and imaging

Double stranded DNA with a length of 10 kbp and with a stock concentration of 500 μg ml^−1^ were obtained from New England Biolabs. DNA solutions (500 pM, 50 pM, 5 pM, 500 fM, 50 fM and 5 fM) were prepared by serial dilution. For fluorescence measurements, DNA was incubated with YOYO-1 (Molecular Probes) at a ratio of five base pairs per molecule. Images and video were collected by a × 60 water-immersion objective and directed to an electron multiplying CCD (emCCD) camera (Cascade II, Photometrics). The CCD camera has a pixel size of 16 μm, however, when used in conjunction with the × 60 objective, the final effective pixel size was 266 nm. In the fluorescence-based measurement, a constant AC field was applied to the outer gold layer of the nanopipette (no DC phase). The pipette was filled with the same buffer used in translocation recordings; however, it should be noted that the filling of the pipette with buffer does not affect the DEP forces as the gold electrode and the maximum field gradients exist just outside the nanopipette's tip.

### Nanopipette fabrication

Nanopipettes were fabricated using a P-2000 laser puller (Sutter Instrument Co.) from quartz capillaries with an outer diameter of 1.0 mm and an inner diameter of 0.5 mm (QF100-50-7.5; Sutter Instrument Co). Nanopipettes were fabricated using a two-line protocol: (1) HEAT: 575; FIL: 3; VEL: 35; DEL: 145; PUL: 75, followed by (2) HEAT: 900; FIL: 2; VEL: 15; DEL: 128; PUL: 200. It should be noted that the above parameters are instrument specific and were optimized to yield 25 nm openings at the tip of the nanopipette. Pipettes were then sputter coated for 60 s to produce a 5 nm coating of gold (Quorum Technologies; Q150R S) and used within several weeks of coating. In rare cases (approximately one in twenty pipettes) gold would delaminate from the pipette and this was observed optically by the DNA being attracted upstream from the tip to where the gold layer was still intact. It was more likely to see the delamination of gold from the second gold electrode: a glass slide coated with 5–10 nm of gold. The conical geometry of the pipette may have attributed to the stability of the gold, which we also observed to increase over time. In cases where pipettes had to be used immediately after gold coating, a thin layer of chromium (2 nm) could be used to increase the level of gold adhesion. Although thicker gold layers were initially tested and proved successful for DEP trapping, thinner gold layers were preferred since longer deposition times had a higher probability of blocking the pore. As for the lower limit of gold deposition, sub-5-nm gold layers had a lower success rate which was due to the lack of conformal coating and/or higher electrical resistance.

### Single channel recordings

The ionic current was measured using an AxoPatch 200B patch-clamp amplifier (Molecular Devices, USA) in voltage clamp mode. The signal was filtered using a low-pass filter at 10 kHz and digitized with a Digidata 1,440 at a rate of 111 kHz and recorded using WINWCP software. WINWCP was used instead of pClamp because it allowed for synchronized triggering of both the AC and DC components. Data analysis was carried out using a custom-written MATLAB analysis routine. The baseline current was calculated via moving window for every data point. Event widths (dwell time) were obtained by measuring the full width half maximum of the current reduction. Current drop was calculated as current peak maximum after subtraction of the baseline current.

### Numerical simulations

FEM computational models of the DEP experiments were created using COMSOL Multiphysics 4.3a along with the AC/DC module. An electrostatic DC approximation was used since the feature sizes of the nanopipette were significantly smaller than the wavelength of the applied AC field. In addition, a 2D-axisymmetric model was used to approximate the nanopipette at normal incidence to the planar electrode (in contrast to the actual 60° angle in experiments). COMSOL Multiphysics 5.0 along with the CFD module was used for ETF simulations, which are further discussed in the Supplementary Information. The threshold |∇|*E*|^2^| was determined from calculating a threshold force for trapping a particle of radius *R* required to overcome Brownian motion, which is given as 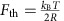 where *k*_B_ is the Boltzmann constant and *T* is the temperature of the particle/solution (298.15 K for all calculations). For the particle radius *R*, the hydrodynamic radius *R*_H_ for 10 kbp DNA was calculated to be 207 nm from the Einstein–Stokes equation^12^,^38^: 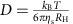, where *D* is the diffusion constant for 10 kbp DNA (1.05 × 10^−8^ cm^−2^ s) and *η*_s_ is the viscosity of the solvent (water, 1.002 mPa s).

## Additional information

**How to cite this article:** Freedman, K. J. *et al*. Nanopore sensing at ultra-low concentrations using single-molecule dielectrophoretic trapping. *Nat. Commun.* 7:10217 doi: 10.1038/ncomms10217 (2016).

## Supplementary Material

Supplementary Information Supplementary Figures 1-15, Supplementary Notes 1-9 and Supplementary References

## Figures and Tables

**Table 1:** Comparison between methods to control capture rate.

Nanopore capture rate enhancement technologies				
Pore type	Mechanism	Factor increase	Event rate	References
α-Hemolysin	Electrostatics at pore	10	0.1 s^−1^ nM^−1^	14,15
Solid-state	Electric field enhancement	40	263 s^−1^ nM^−1^	12
Nanopipette	Dielectrophoresis	80	1,040,000 s^−1^ nM^−1^	This study
