# Supplementary material for: Nanopore sensing at ultra-low concentrations using single-molecule dielectrophoretic trapping
Source: Nat Commun. 2016 Jan 6;7:10217. doi: 10.1038/ncomms10217 (PMC4729827; doi:10.1038/ncomms10217)
Supplement: Supplementary Information — Supplementary Figures 1-15, Supplementary Notes 1-9 and Supplementary References [file ncomms10217-s1.pdf]

Supplementary Figures

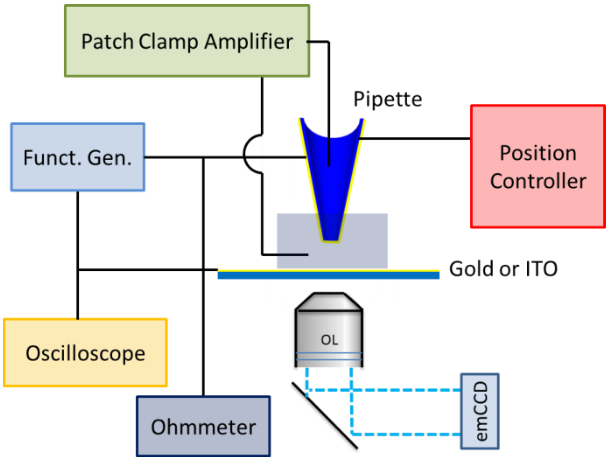

Supplementary Figure 1. Schematic diagram of the system used to locate the position of the pipette tip relative to the gold-coated glass slide.

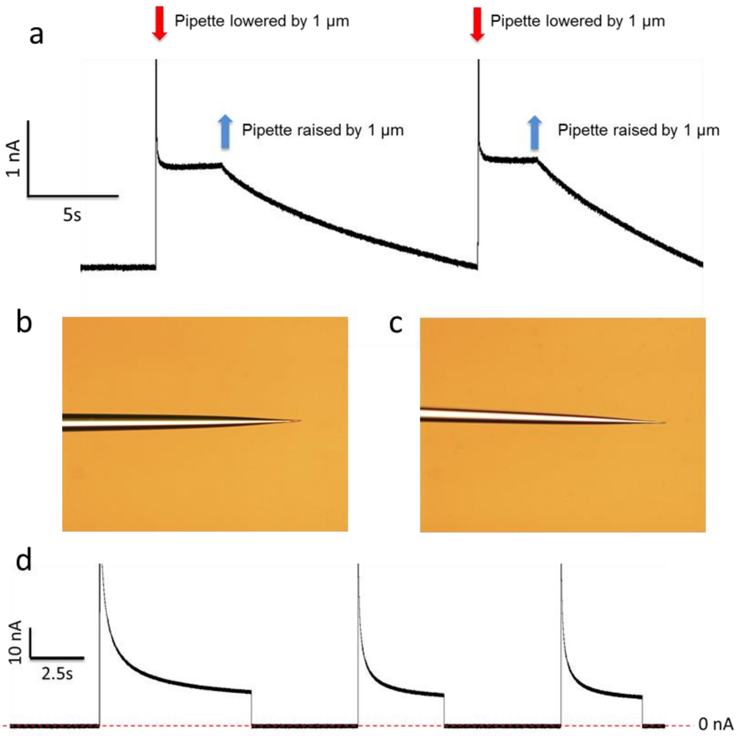

Supplementary Figure 2. Tip positioning relative to a gold-coated glass slide. (a) Ionic current traces observed when a +500 mV was applied across the Ag/AgCl electrodes and the pipette was lowered using micromanipulators. Optical micrographs of the pipette before (b) and after (c) making contact with the gold-coated glass slide. (d) Electrical current measured between the gold coated slide and the gold coated pipette using a 10 mV voltage bias.

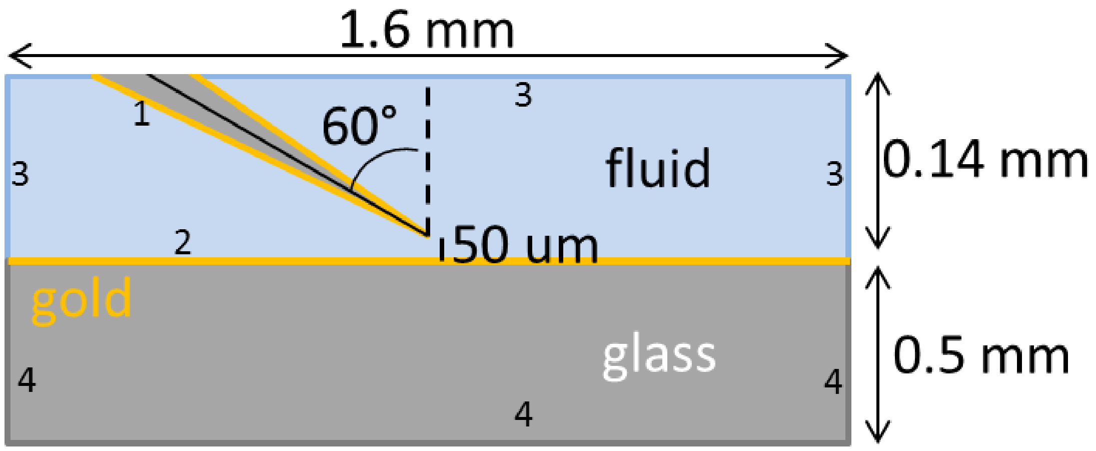

Supplementary Figure 3. Schematic of the modelling domain, geometry, and boundaries. The modelling of our experimental conditions in Figure 3 (σ = 16 mS/m, f = 1 MHz, $V_{pp} \geq$ 10V) show that the applied AC signal creates an electrothermal flow with velocities ≥100 µm/s within 20 µm of the nanopipette tip with peak velocities near the tip (≤10 µm) also comparable to the data in Figure 3f.

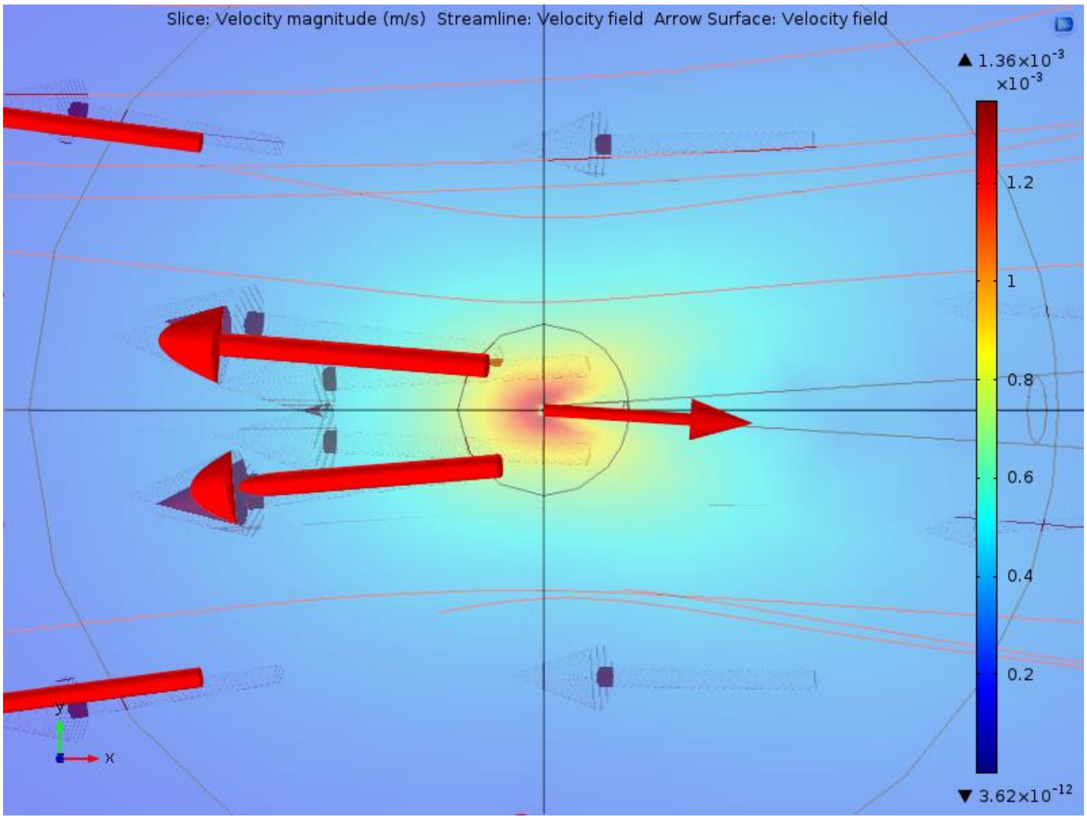

Supplementary Figure 4. A 2D mapping of the flow velocity magnitude. The data is in the xy plane at z = 0, which is level with the tip of the nanopipette. The red lines are stream lines showing the direction a particle in the fluid would flow, and the red arrows give the flow direction.

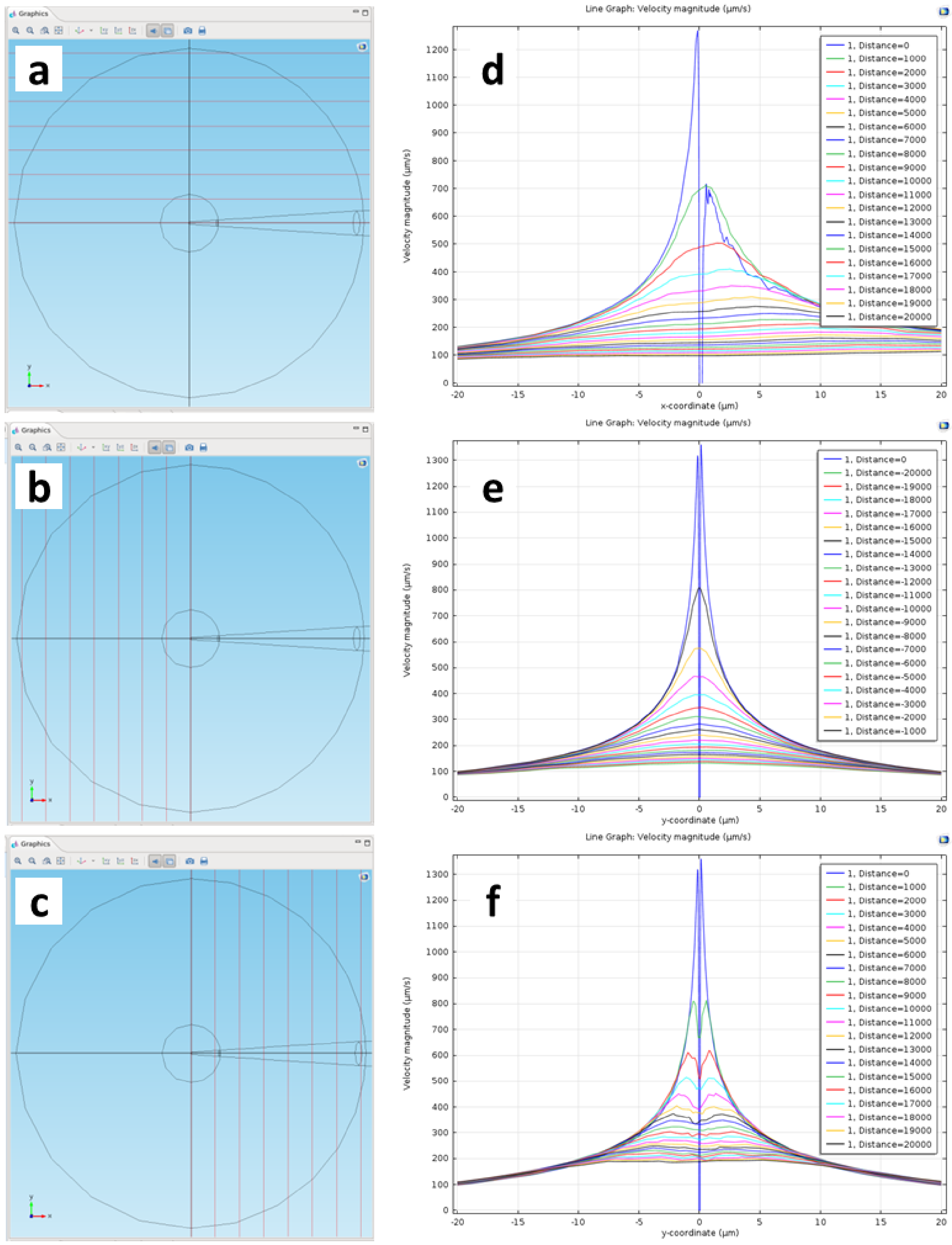

Supplementary Figure 5. The velocity magnitude at various distances from the nanopipette in the x-y plane at z = 0. (a)-(c) show the lines along which the data in (d)-(f) are plotted. The line separation is 1 µm. The velocity is mostly in the –x direction.

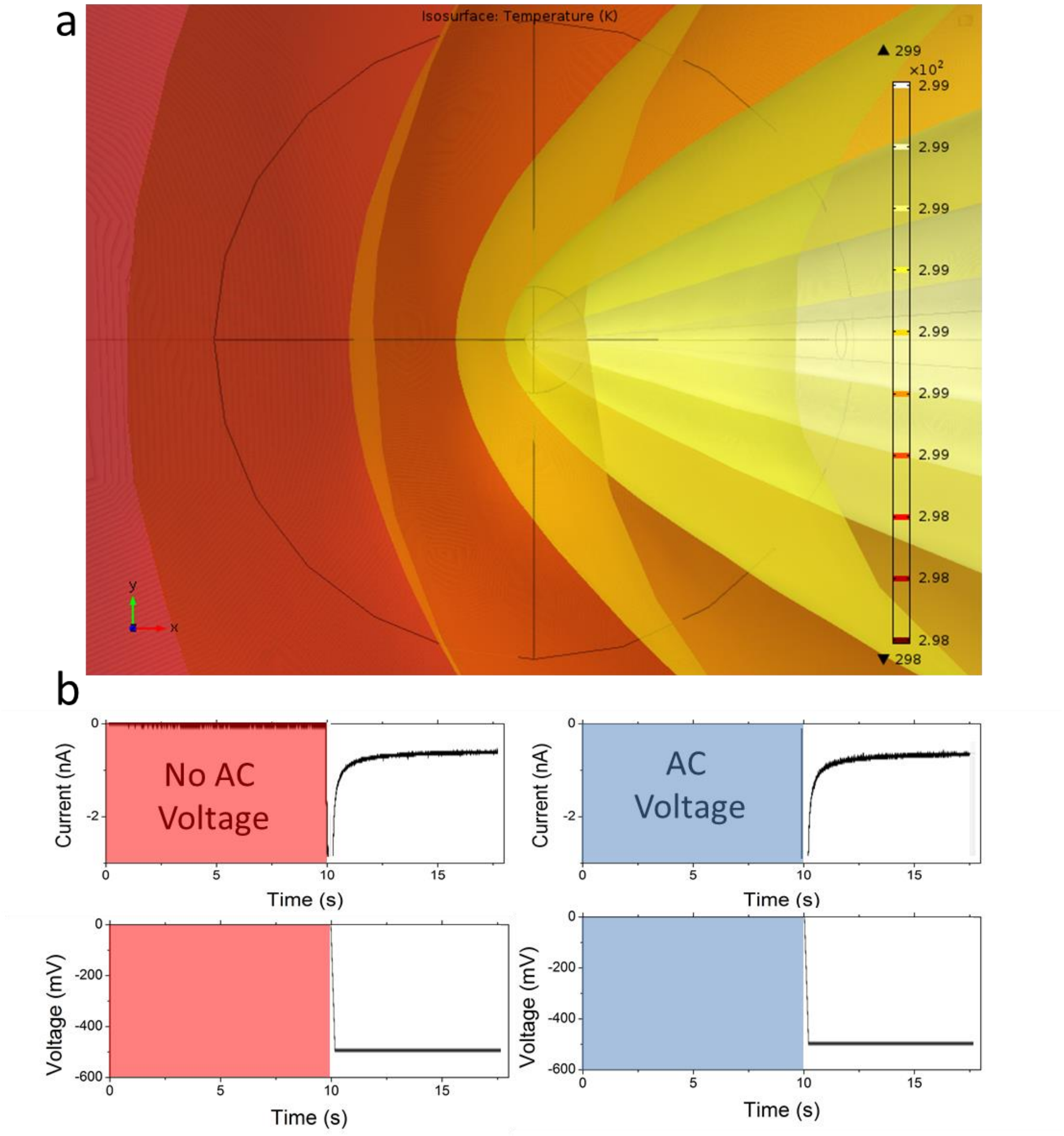

Supplementary Figure 6. (a) FEM model temperature plot showing minimal heating effects by the application of a 10 V, 1 MHz AC voltage. (b) Ionic current measurements with and without an AC voltage applied to the outer gold layer of the pipette.

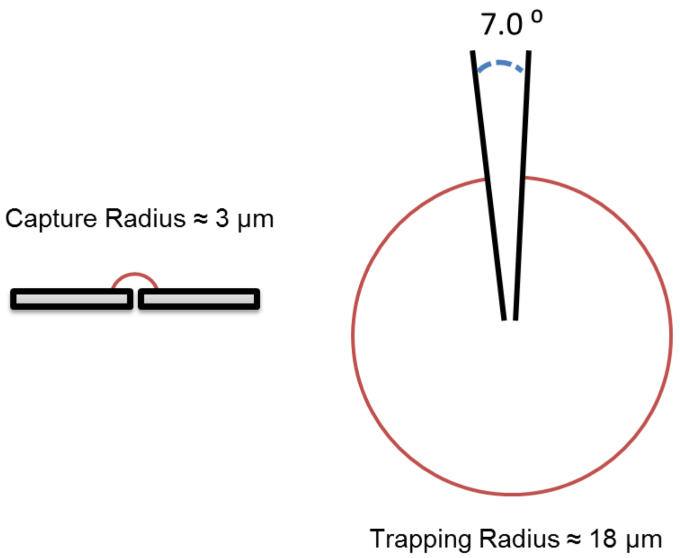

Supplementary Figure 7. Comparison between the capture radius using solid-state pores (electrophoretic capture mechanism) and metallized nanopipettes (using a DEP trapping mechanism). The sizes of the capture radii are to-scale.

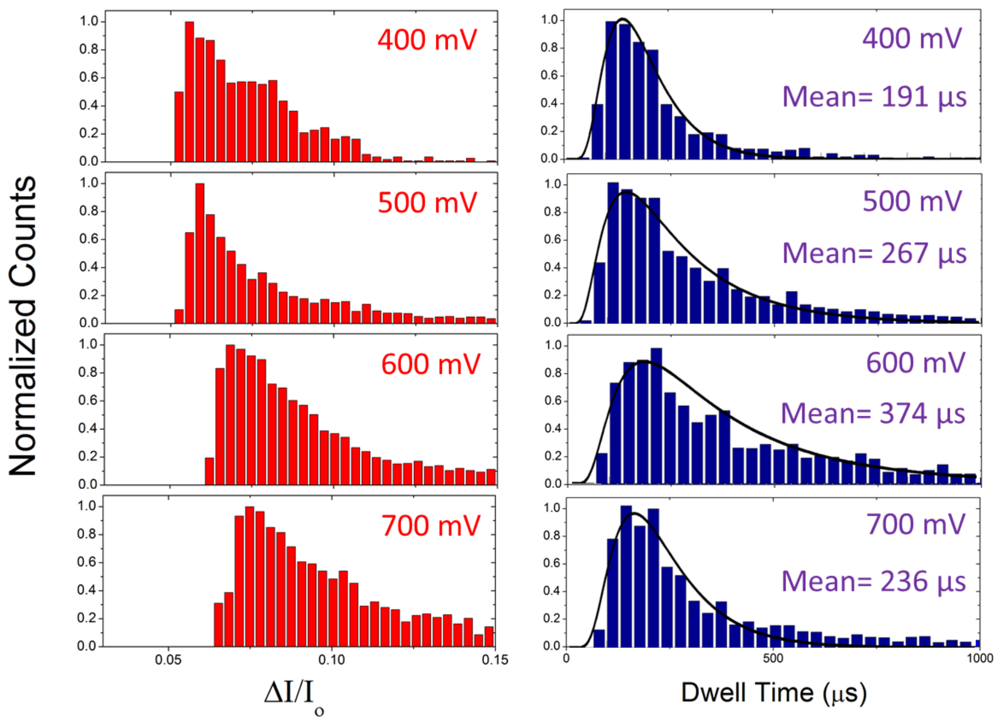

Supplementary Figure 8. Histograms for the normalized spike amplitude ($\Delta I/I_o$) and the dwell time for $|\Delta V|$= 400, 500, 600 and 700 mV. The dwell time was fitted using the Ling-Ling model and the mean is printed for each voltage condition[1]. The number of events for each voltage are n=970, 2328, 3304, 2605 for 400, 500, 600, and 700 mV, respectively.

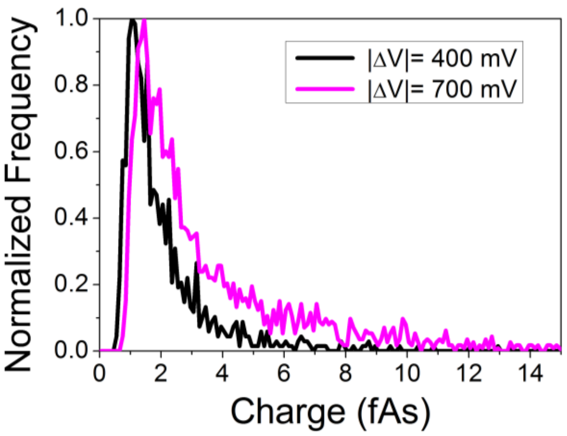

Supplementary Figure 9. Normalized histogram of the equivalent charge (the area under each event signature) at the lowest and highest voltages tested.

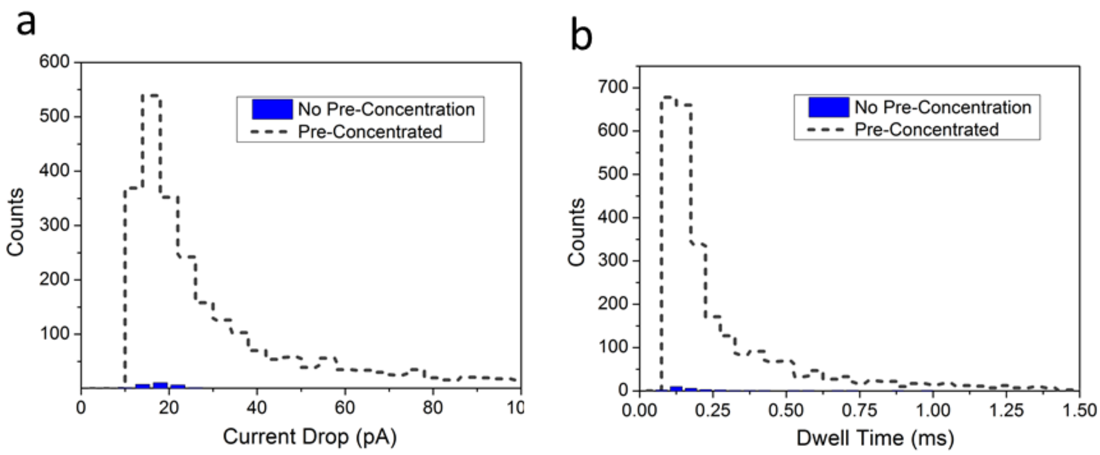

Supplementary Figure 10. Time-normalized translocation data with and without DEP pre-concentration. The data was time-normalized (recording time=180 s) and plotted on a linear scale to clearly show the magnitude of the DEP enhancement. The number of events detected while using DEP to pre-concentrate the DNA was 2715 while without trapping resulted in only 34 events.

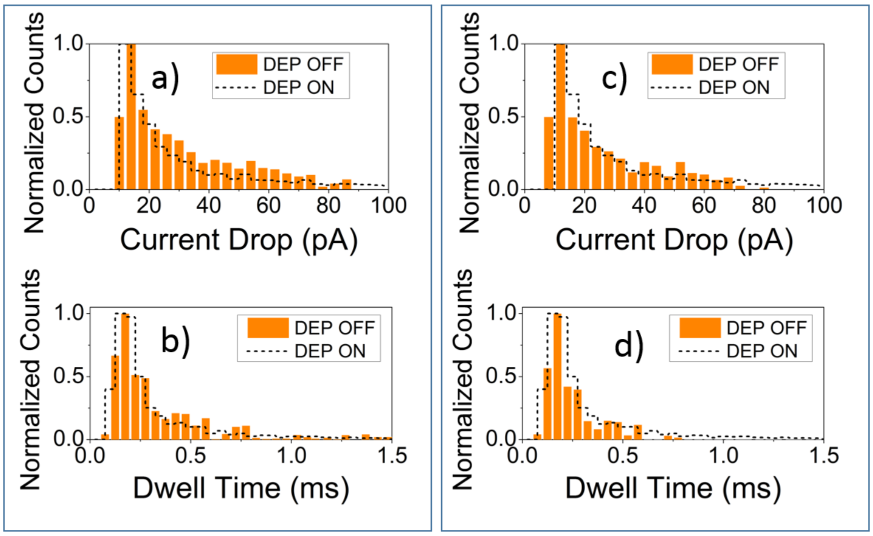

Supplementary Figure 11. Dwell time and current drop histograms for conditions where DEP was used prior to recording translocations and when no DEP was used. This plot also demonstrates the pipette variability in the data since two pipettes were used to collect this data (a-b, and c-d were from different pipettes). The data presented here supplements the data in the main text where there are small variations in the tail of the distribution. Several pipettes were used during the course of this study and variations in the distributions were found which reflect the deviation caused by using different pipettes. The main attributes of the distributions remain unchanged with the peak of the distributions staying constant. As discussed in the main text, larger current drop events occur when DEP is used to concentrate DNA before a recording however these events make up a small fraction of the events.

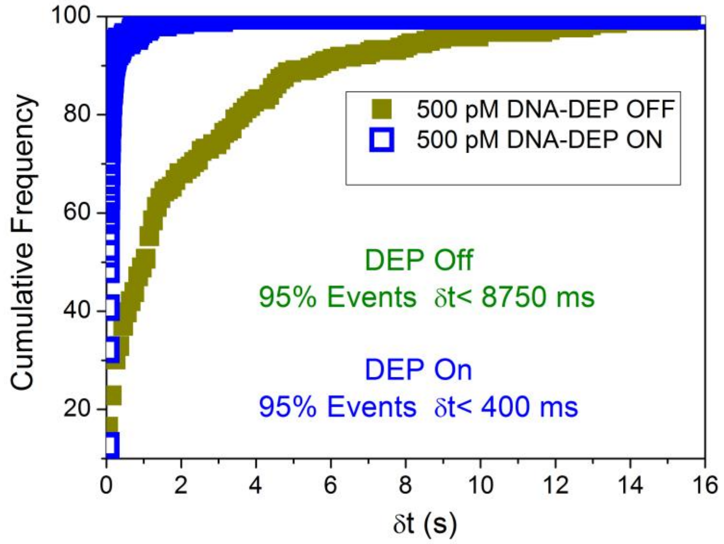

Supplementary Figure 12. Inter-Event Times for DEP Pre-Concentration. Cumulative inter-event times for a 500 pM 10 kbp DNA sample with and without DEP pre-concentration. Event analysis of translocation events acquired through DEP-based pre-concentration at V_{pp}= 12 V_{pp}, f_{AC} = 1 MHz, and d_{gap}=50 µm.

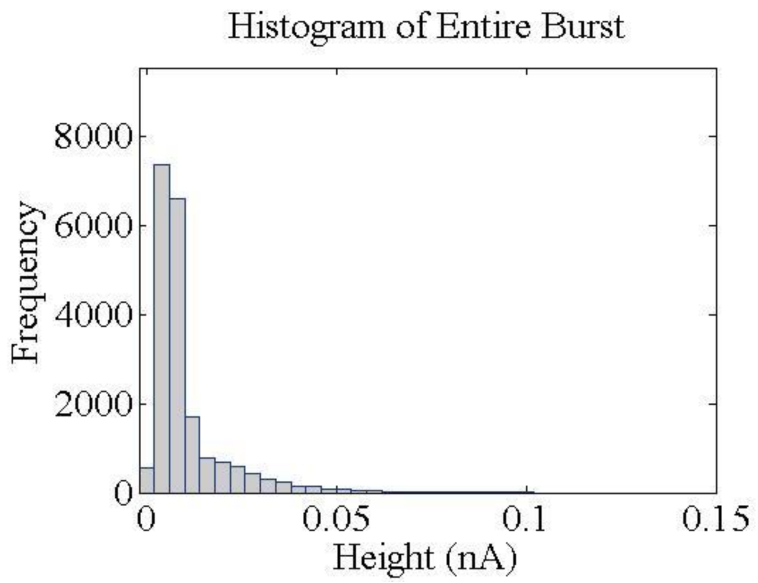

Supplementary Figure 13. All-points histogram of events only (no baseline ionic current data). Events were recorded using a voltage bias of 500 mV and with no DEP prior to the recording (n=1384 events).

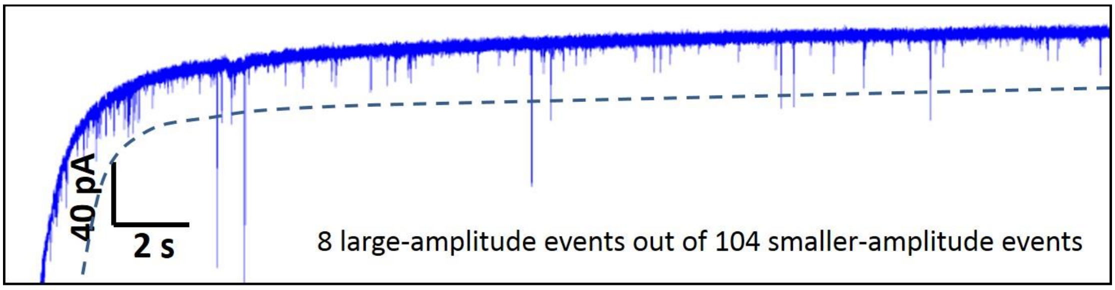

Supplementary Figure 14. Expanded view of the current trace in Figure 4a. A dotted line is used to discriminate candidate DNA aggregates which is set at 2× the peak current drop of all DNA events.

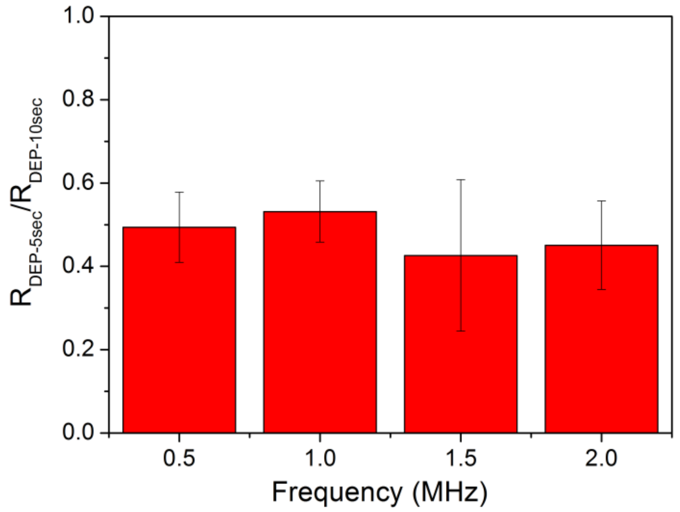

Supplementary Figure 15. The capture rate for 5 seconds of DEP trapping divided by the capture rate for 10 seconds of DEP trapping across four DEP trapping frequencies.

Supplementary Notes

Supplementary Note 1

Nanopipette Tip Positioning The nanopipette was positioned relative to the gold-coated glass slide using two independent methods. The first was based on the change in nanopore conductance upon lowering the tip towards the glass slide until contact was made. The ionic current was measured across the Ag/AgCl electrodes in voltage clamp mode using pClamp software (Molecular Devices). A full system diagram of our experimental setup is shown in Supplementary Figure S1. Using micromanipulators to control the nanopipette's position, the tip was lowered in steps of 100 µm until a deflection in the ionic current was observed. Following this, the tip was raised by the same amount and the step size was reduced to 50 µm and the same process was repeated. The step size was reduced until we could reliably sense the position of the counter electrode using a step size of 1 µm. Supplementary Figure S2a shows how the ionic current changes with consecutive up-down-up-down tip movement. The current increases due to the increase in surface ion concentration within the double layer immediately adjacent to the gold surface. This effect is particularly apparent because of the low salt concentration that is used (1 mM). Raising the tip causes the ionic current to return to baseline conditions which shows that the size of the tip is not altered by the process. The same pipette used in Supplementary Figure S2a was also imaged before and after the positioning protocol and no major tip changes were observed.

The second method of locating the surface was based on the resistance change measured between the gold coating on the pipette and the gold on the coverslip surface. The pipette was lowered using the same protocol as above however an ohmmeter was used measure the resistance change associated with the two electrodes coming into contact. Typically the resistance between the tip and surface in a 1mM salt solution was 16-18 MOhm. Once the tip is lowered to be in contact with the gold–coated slide, the resistance measured by the Ohmmeter was 0.3 kOhm. The electrical current between the two gold electrodes was also measured while a bias of 10 mV was applied. Electrical current is able to flow only when the two gold electrodes contact (Supplementary Figure S2d).

Supplementary Note 2

The Counterion Fluctuation Model While the Clausius-Mossotti factor (CMF) is a good estimate for dielectric particles such as polystyrene beads, recent literature has suggested that the assumption of redistribution of charges is not appropriate for molecules such as DNA because the negative charges are fixed to the molecule's backbone. Instead the counterion fluctuation (CIF) model has been proposed as the alternative for describing the true nature of the polarizability of these molecules. Specifically, that the redistribution of the counterions from the solution which surround the molecule near charged sites are the most critical to contributing the polarizability of DNA.

Prior works have referred to experimentally measuring a dielectric decrement, $\Delta\epsilon_{DNA}$, for specifically the DNA and the experiments in those works. For 12 kbp DNA (0.9 mg/mL) in 8 mS/m conductivity solution at 2 MHz, the dielectric decrement is ~4. Following the trends in the previous work, it was estimated that for conditions of 10 kbp DNA at 16 mS/m conductivity (1 mM KCl) and 1 MHz the dielectric decrement would be $\Delta\epsilon_{DNA}$~5 (dimensionless). Using Avogadro's number (N_{Av}), dielectric constant of the solution (ϵ_{1mM} = 78.4), vacuum permittivity (ϵ_0 = 8.85e-12 F/m), Boltzmann's constant (k_B), and the temperature (T = 273.15 + 25 K), a polarizability (in F m^2) of the DNA in prior works was calculated through the relationship:

$$\alpha_{DNA}(c) = \frac{3\Delta\epsilon_{DNA}\epsilon_0}{10^{-12}N_{Av}c} \qquad (1)$$

where c is the concentration in fM. This method yields a DNA polarization that is dependent on the concentration of the DNA. The concentrations in the aforementioned works were all larger (~100 nM or higher) than for this current work, and yielded polarization values comparable to other literature[2]. However, for our experiments, this calculation method means that the polarizability of the DNA increases to an unrealistic value, and this approximation is not likely correct for the extreme parameters of this current work. For example, the polarizability of our DNA at 100 nM and 5 fM (the lowest concentration used) would be 2.2e-30 F m^2 and 2.2e-23 F m^2, respectively.

The underlying assumption for this equation is that there are more ions in solution than there are DNA molecules. In other words, more counterions per DNA means that the DNA is more polarizable. But this effect must eventually saturate. But for low concentrations of DNA in high concentrations of buffer like in this current work, which is unique from previous work, we would not expect this trend to hold. Since there are 2 negative charges per base pair of DNA[3], we can approximate that there are 20,000 necessary counterions per 10 kbp DNA strand. A general rule of thumb is:

$$\frac{C_{ions}}{C_{DNA}} \gg N_{ions/DNA} \qquad (2)$$

where $N_{ions/DNA}$ is the number of counterions from the solution required to "saturate" the polarizability of the DNA. For 10 kbp DNA, we would expect $N_{ions/DNA}$ ~20,000, which holds true for all experiments in this work. For the concentrations of ~1 mM KCl and ~100 pM or less DNA (as often used in this work) the factor is 10^7 which is much greater than 10^4. Even the 10 nM DNA solution, which is the highest used concentration, and only for fluorescence based measurements, meets this qualification.

Calculating the polarizability for the CIF model requires many parameters. The first are the valence of each counterion (1 for KCl solutions) (z), the average distance in m between the charged sites (b_{CIF}), and the elementary charge (q) in Coulombs. The charge density parameter (ξ) is given by:

$$\xi = \frac{q^2}{4\pi\epsilon_{rm1mM}\epsilon_0 k_B T\, b_{CIF}} \qquad (3)$$

and for a 1 mM KCl solution at 25° C, ξ = 4.14 (a dimensionless quantity).

Two additional parameters are necessary for the calculation of the Debye screening length, the diffuse ion concentration (C_i = 1 mol / m^3 for 1 mM KCl) and the concentration of phosphate

groups on the DNA (C_p = 2.72 mol / m^3), which corresponds to the ion concentration near the DNA[4, 5]. κ_S is the reciprocal of the Debye screening length (λ_D) and is given by:

$$\kappa_S = \sqrt{\left(\frac{N_{Av}q^2}{\epsilon_{rm1mM}\epsilon_0 k_B T}\right)\left(C_i z^2 + \frac{C_p}{\xi}\right)} \qquad (4)$$

where κ_S was found to be 9.47e7 m^-1 and $\lambda_D$was found to be 10.6 nm. A_{st} is the stability factor of the ionic phase and includes mutual repulsion between fixed charges on the backbone and the effect of Debye screening. It is a dimensionless quantity described by:

$$A_{st} = \frac{1}{1-2(z\,\xi-1)ln(\kappa_S b_{CIF})} \qquad (5)$$

and found to be A_{st} = 0.037 for our work. L_S is a subunit length of the DNA over which a subunit polarization is calculated (a few lines below) using the "Manning-Mandel-Oosawa" model and was calculated to be L_S = 74.2 nm through the following relation:

$$L_S = \sqrt{\frac{9\Delta\epsilon_{DNA}}{\pi\epsilon_{rm1mM}(z\,\xi-1)A_{st}N_{Av}C_p b_{CIF}}}. \qquad (6)$$

(Note that L_S is dependent on the dielectric decrement) The fraction of condensed counterions (ϕ_c) was also calculated to be 0.76 (and is dimensionless) through the relation:

$$\phi_c = 1 - \frac{1}{z\,\xi}. \qquad (7)$$

Finally, the number of condensed counterions (n_{cc}) was also calculated to be 325 for the length L_S using the equation:

$$n_{cc} = \frac{\phi_c L_s}{z\, b_{CIF}}. \qquad (8)$$

The number of condensed counterions for the entire DNA length could be calculated by multiplying n_{cc} by the ratio of the full length to the subunit length (l_{DNA}/L_s where l_{DNA} = 3.4 µm for 10 kbp DNA). This yields a total of 14,900 condensed counterions for each entire DNA, which is close to the given approximation of 20,000 counterions for the entire DNA macromolecule. When normalized by the fraction of condensed counterions, ϕ_c, the approximation is even closer (19,700). Therefore, we believe that this new approximation is effective, and it is valid to assume that the DNA is saturated with counterions in the solution; therefore, the polarizability of these DNA cannot be increased by decreasing the DNA concentration (as would be the case using the equation given in previous work with high DNA concentrations relative to the ion solution).

Continuing with the CIF model calculation, the subunit polarization (α_s), which is the polarization for subunit length of the DNA, is defined by:

$$\alpha_s = \frac{z^2q^2L_S^2 n_{cc}A_{st}}{12\, k_B T} \qquad (9)$$

and was calculated to be 3.48e-32 F m^2. The total polarization (α_{CIF}) for the entire 10 kbp DNA can be calculated by multiplying α_s by the ratio of the full length to the subunit length (l_{DNA}/L_s) as above, which yields a DNA macromolecule polarizability of 1.59e-30 F m^2. This polarization value matches expectations based on other literature[2]. It is also independent of the DNA concentration.

In order to calculate an approximate threshold force required to trap a 10 kbp DNA for our experiments, we estimated the DNA size using the hydrodynamic radius. For this calculation, we needed two additional parameters: D_i = 1.5e-12 m^2/s is the diffusion constant for 10 kbp DNA at room temperature and η_s = 1.002e-3 Pa s is the viscosity of the medium (water). The hydrodynamic radius (r_H) is the effective size of the DNA assuming the worm-like chain model, which would be how the DNA is arranged in solution, and was calculated to be 207 nm through the equation:

$$r_H = \frac{k_B T}{6\pi\eta_s D_i} \qquad (10)$$

This was then used in the same Brownian motion threshold force equation given by Pohl[6]:

$$F_{th} = \frac{k_B T/2}{r_H} \qquad (11)$$

The threshold force was calculated to be 9.92 fN for the 10 kbp DNA.

To relate these results to our COMSOL modelling of the DEP forces, a threshold electric field intensity gradient ($\nabla|E|^2_{th}$) was also calculated to be 2.49e16 V^2/m^3 through the relationship:

$$\nabla|E|^2_{th} = \frac{4\,F_{th}}{\alpha_{CIF}} \qquad (12)$$

for the 10 kbp DNA. On the logarithmically scaled plots, $Log_{10}(\nabla|E|^2_{th})$ = 16.4. For the 10 V model, 50 um electrode gap, this $\nabla|E|^2_{th}$outlines a trapping volume that extends 4.4 µm upward from the pipette center and 5.7 µm outward (on each side) from the face of the pipette (in the 2D axisymmetric model). For both the trapping volume also does extend somewhat (<1 µm) below the face of the pipette, as previously discussed, so the entire trapping volume is larger than a hemisphere.

Supplementary Note 3

Electrothermal Flow Modelling In DEP experiments, electrokinetic flow (electroosmotic, electrothermal, etc.) must be considered. For the parameters of our DNA experiments (σ = 16 mS/m, f = 1 MHz, $V_{pp} \geq 10$V), we expect electrothermal flow to be a significant contributor to the movement of the DNA macromolecules and therefore the capture rate enhancement. Electrothermal flow modelling was performed in full 3D using COMSOL Multiphysics™ 5.0 along with the Computational Fluid Dynamics module in order to further understand the observed electrokinetic flow and the mechanism for the capture rate enhancement due to DEP and the induced Joule heating in the fluid from the applied AC field. The model was constructed by the coupling of Electric Currents, Heat Transfer, and Laminar Flow through the following equations. For an AC system, Joule heating creates an inhomogeneous temperature throughout the fluid according to:

$$k\,\nabla^2 T + \sigma\, E_{rms}^2 = \rho\, C_p \boldsymbol{u} \cdot \nabla T \qquad (13)$$

where k, T, σ, ρ, and C_p are the thermal conductivity, temperature, conductivity, density, and heat capacity at constant pressure of the medium, respectively, and u is the velocity of the fluid. E_{rms} is the electric field generated due to the AC signal applied between the two electrodes. In this case, V_{rms} was defined in the model to be $V_{pp}/\sqrt{2}$ and was applied to the surface of the nanopipette (boundary 1) as shown in Supplementary Figure S3, while boundary 2 was grounded. The Electric Currents physics was used to calculate the electric field for the entire region of the fluid, and the resulting field induced a temperature gradient in the fluid according to:

$$\rho\, C_p \boldsymbol{u} \cdot \nabla T = \nabla \cdot (k\, \nabla T) + Q_e \qquad (14)$$

where $Q_e = \sigma E_{rms}^2$ and is the power generated by the electric field. The conductivity, σ, was measured to be 0.145 mS/m for DI water and 16 mS/m for the 1 mM KCl solution and was applied to the model. The values for k, ρ, and C_p were taken by the model from the temperature-dependent curves for water given in COMSOL. The gold material was applied as a boundary material to both boundaries 1 and 2 because the gold thickness of 5-20 nm is much less than the other dimensions in the model, and these boundaries were described by a diffuse boundary condition:

$$-\boldsymbol{n} \cdot (-k\, \nabla T) = \varepsilon_s \sigma (T_{amb}^4 - T^4) \qquad (15)$$

where **n** is the normal vector to the boundary, ε_s is the surface emissivity, and T_{amb} is the ambient temperature of 25°C (298.15K). The surface emissivity of the gold was approximated to be ~0, which is a reasonable approximation for a foil or thin film. The outside of the fluid was also approximated as a diffuse surface with a surface emissivity of 0.96. The outside surfaces of the glass slide (boundary 4) were held at a constant ambient temperature of 25°C. The Heat Transfer physics was used to calculate the temperature gradient in the fluid. Both the temperature gradient and the electric field resulting from Joule heating contribute to a body force in the solution described by:

$$\langle \boldsymbol{f_E} \rangle = -\frac{1}{2}\left[\left(\frac{\nabla\sigma}{\sigma} - \frac{\nabla\varepsilon}{\varepsilon}\right) \cdot \boldsymbol{E}\ \frac{\varepsilon \boldsymbol{E}}{1+(\omega\tau)^2} + \frac{1}{2}\boldsymbol{E}^2 \nabla\varepsilon\right] \qquad (16)$$

where ε is the permittivity of the fluid, ω is the frequency of the applied signal, and τ is the charge relaxation time of the solution (ε/σ). Using the relationships:

$$\frac{1}{\varepsilon}\frac{\delta\varepsilon}{\delta T} = \alpha = -0.004\ \mathrm{K}^{-1}$$

$$\frac{1}{\sigma}\frac{\delta\sigma}{\delta T} = \beta = 0.02\ \mathrm{K}^{-1}$$

which describe the dependence on temperature of the permittivity and conductivity of the aqueous solution, the time-averaged body force equation can be rewritten as:

$$\langle \boldsymbol{f_E} \rangle = \frac{1}{2}\left[(\alpha - \beta)(\boldsymbol{\nabla} T \cdot \boldsymbol{E})\frac{\varepsilon \boldsymbol{E}}{1+(\omega\tau)^2} + \frac{1}{2}\boldsymbol{E}^2 \varepsilon \alpha \boldsymbol{\nabla} T\right] \quad (17)$$

A Volume Force node was added to the Laminar Flow physics, and the vector components of this equation were entered to generate the electrothermal flow in the fluid described by the incompressible flow Navier-Stokes equation along with mass conservation:

$$0 = \boldsymbol{\nabla} \cdot [-p\boldsymbol{I} + \mu(\boldsymbol{\nabla u} + (\boldsymbol{\nabla u})^T)] + \langle \boldsymbol{f_E} \rangle \quad (18)$$

$$\rho \boldsymbol{\nabla} \cdot \boldsymbol{u} = 0 \quad (19)$$

where p is the pressure in the fluid, μ is the dynamic viscosity of the fluid, and **u** is the velocity in the fluid. A no-slip condition (**u** = 0) was used on boundaries 1 and 2; however, since boundary 3 was exposed to air, a slip condition was used.

Supplementary Note 4

Temperature Effects Temperature increases of the fluid near the nanopipette due to Joule heating were investigated by experimental and theoretical methods. Experimentally, the effects of heating were tested by applying an AC voltage for 10 seconds, and then turning on the DC component. Applying the DC voltage typically results in a capacitance spike which lasts ~100 ms. If heating was significant, it would be expected that the pore conductance would take a longer time to reach baseline conditions[7, 8]. The results show that the ionic current is stable and not elevated to a higher conductance level, especially not during the 8-10s recording window where translocation events are observed. Since the flow of ions through the pore is temperature sensitive, any local temperature changes would be evident by the ionic current. The temperature was also solved for using FEM models, which showed minimal temperature increases ($\Delta T= \sim 1$ ˚C) for the AC voltages used in these experiments (Supplementary Figure S6).

Supplementary Note 5

Calculation of Trapping Volume The calculation of the capture volume using conventional nanopore techniques was conducted using the most generous value for the capture radius using a solid-state nanopore (~3 µm)[9] but in reality the capture radius will be smaller for 10kbp DNA[10], Supplementary Figure S7. Due to the geometry of the solid-state pore, molecules are only captured from one side the planar membrane leading to a capture volume modelled as half the volume of a sphere: $V = \frac{1}{2}\left(\frac{4}{3}\pi r^3\right)$. Since a 3 µm capture radius corresponds to a 56 µm^3 capture volume and a 1 nM DNA solution has 0.6 DNA molecules per um^3, the number of DNA molecules per capture volume is approximately 34 molecules. Furthermore, at 5 fM DNA, there are 1.7 ×10^{-3} molecules per capture volume which makes sub-pM DNA detection extremely inefficient using current nanopore technology.

For the nanopipette, the trapping volume was calculated as the volume of a sphere minus the volume of a cone: $= \frac{4}{3}\pi r^3 - \pi r_c^2 \frac{h}{3}$, where h is the trapping radius (assuming the tip is in the center of the sphere) and r_c is the radius of the pipette at the distance h upstream from the tip. The value of r_c is calculated as $r_c = h \tan\left(\frac{7}{2}\right)$. The trapping volume assuming a radius of ~18 µm, is 24,406 µm^3. The trapping radius of 18 µm was used as an approximation for the trapping radius considering DNA tracking data shown in the main text (Figure 3f) which was conducted at 12V_{pp} and 1 MHz. The trapping volume could become bigger or smaller than this depending on the AC voltage being applied to the nanopipette, Supplementary Figure S7.

Supplementary Note 6

DNA Translocations Using Gold-Coated Nanopipettes Prior to DEP trapping experiments, the conditions for electrically sensing DNA were optimized and tested using gold-modified nanopipettes. In these preliminary experiments the distance between the tip and the surface was not controlled and a voltage-dependent analysis of the translocation kinetics was conducted. Quartz nanopipettes coated with gold were filled with 1 mM KCl and lowered into a well electrolyte of the same concentration. The use of 1 mM salt was based on maximizing the signal to noise ratio for the detection of DNA[11]. Using a bias voltage between -400 mV and -700 mV, DNA was transported through pore from the solution outside the pipette to the inside of the pipette. Translocations are therefore driven by electroosmotic flow (EOF) in the opposite direction to the electrophoretic force. EOF-driven translocations are expected at low salt concentrations due to the high surface charge density of the quartz inside the pipette and the gold outside the pipette (both negatively charged surfaces)[12].

Another effect of using [KCl]<0.4 M was that the conduction of ions through the pore is increased during the translocation process[11, 13, 14]. At low salt, the high charge density of DNA in combination with the low bulk ion concentration in solution results in a large counter-ion cloud around the DNA molecules. During translocations, the counter ions experience a strong electric field which strips the ions off the DNA backbone. The ions introduced into the pore by the DNA are greater in quantity that the exclusion of ions due to the DNA itself. Therefore, the increase of ionic charge within the pore yields a conductive spike in the measured current. To date, the voltage dependence of this phenomenon has yet to be studied.

The number of ions which contribute to the conductive ion spikes as a function of voltage is thought to increase with larger voltage until the maximum number of mobile ions are stripped from the DNA backbone. Indeed, the normalized ionic current ($\Delta I/I_o$) increases in magnitude as the applied voltage is increased within the range we studied (Supplementary Figure S8). If the same number of ions were removed from the DNA, the conductive spike amplitude would decrease since the exclusion of ions due to the DNA strand blocking bulk ion flow is known to have a linear increase which would reduce the overall spike amplitude. Instead, we observe an increase in the spike amplitude, the normalized spike amplitude, and the dwell time up until $|\Delta V|$= 600 mV at which point the mean dwell time becomes shorter compared to $|\Delta V|$= 500 mV. Most importantly however is that the peak dwell time for all voltages is approximately the same across voltage with only the variation in the distribution changing. Although DNA configuration may play a role in the time constant responsible for ions being stripped from the DNA, this was ruled out since the same DNA solution was used throughout these experiments. The charge (quantified by the area under the ionic current signature) also shows an overall increase between the lowest and highest voltages tested indicating a net increase in the number of ions being removed from each DNA molecule (Supplementary Figure S9).

Supplementary Note 7

DNA Aggregate Frequency The frequency of DNA aggregates caused by DEP can be obtained by setting a threshold and counting threshold-crossings per unit time. This method lacks accuracy since recordings that were not preceded by DEP also yield threshold crossings (albeit fewer than when DEP was used). Setting a threshold for 2× the peak current drop for all DNA events (with no DEP) was used to give a rough estimate of dimer formation when DEP was used to concentrate DNA molecules. Although large-amplitude events (>40 pA) stand out above the noise, Supplementary Figure S13-S14 shows that there are far greater small-amplitude events.

A better representation of the DNA aggregate frequency caused by DEP is to subtract out the number of events expected to produce a threshold crossing when DEP is not used. Using the same threshold setting as before (2× the peak current drop), the number of threshold crossings was 284 events out of 2328 total events (10.2 %). The same threshold was applied to all the recordings used to create Figure 4 in the main text where V_{pp}= 12 V_{pp}, f_{AC} = 1 MHz, and d_{gap}=50 µm. With these conditions, there were a total of 385 threshold crossings out of 2715 events (14.18%). Since 10.2% events crossed the threshold without DEP, then the percentage of events that crossed the threshold simply by turning on the DEP was approximately 4%. The molecules that produced these large current blockades are likely DNA aggregates that became associated with other DNA molecules while trapped at the tip of the nanopipette.

Supplementary Note 8

DEP Trapping Time Dependence DNA motion was controlled in our experiments using DEP which acted to preconcentrate the DNA within the local area around the nanopipette tip as well as electrokinetically (through EOF) by driving the DNA through the nanoscale opening in the tip. The dominant force which influenced the capture rate is shown to be the preconcentration phase of the experiment. Throughout our experiments, a 10 second long DEP trapping period was used to preconcentrate the DNA. Since the amount of DNA which is trapped scales linearly with the time of DEP trapping, we would expect that 5 seconds of DEP trapping would result in half the capture rate during the translocation phase of the experiment. Indeed, the ratio between capture rates measured with a 5 second versus a 10 second DEP trapping time ($R_{DEP\text{-}5sec}/R_{DEP\text{-}10sec}$) produced an average ratio of 0.476 across the four frequencies used for DEP trapping, Supplementary Figure S11-12 and S15

Supplementary Note 9

Capture Rate Increase over Multiple Cycles The question of whether the DNA diffuses within the DC-phase capture radius or adsorbs to the pipette surface nevertheless still persists. If DNA is located close to the nanopore during a DEP cycle, then the timescale in which DNA could be re-captured by the DC potential depends on how far diffusion can displace the DNA after the AC voltage is turned off. The recapturing of DNA using biphasic voltage pulses was shown previously by Gershow and colleagues[15]. In this study DNA could be recaptured after a 32 ms delay period with a probability of ~0.4. Despite the fact that this study kept a +120 mV potential applied to the pore which would actively drive DNA away from the pore during the delay period (whereas this study applied no potential post-DEP trapping), diffusion alone is expected to displace the DNA over time. Assuming the capture radius during DC field-driven translocations is at most 3 µm from the pore[9, 16], then DNA should diffuse out of the capture radius in 3.21 s, (calculated using the equation for mean squared displacement) which is much shorter than the >8 minutes (20s × 25 cycles = 8.3 min.) of enhancement observed here. In order to explain the post-DEP enhancement observed in Figure 5a, it is believed that DNA becomes adsorbed onto the gold surface during DEP trapping and randomly desorbs over time. The maximum capture rate occurred after seven cycles where DEP was used to pre-concentrate the DNA and an enhancement factor (EF) observed over the baseline capture rate was EF = 160.

A key finding of our trapping studies is that DNA is accumulates within the capture radius of the nanopipette. This can be said with certainty by the fact that the preconcentration of DNA took several cycles to increase to its maximum level of enhancement. The first 10 seconds of DEP preconcentration, DNA is attracted towards the tip and only a fraction of the DNA is translocated. A portion of the trapped DNA becomes adsorbed onto the pipette's outer surface and contributes to the capture rate measured in subsequent cycles. The adsorption and random desorption from the pipette surface is essential to DNA sensing in the fM concentration range. Furthermore, we observed that the stronger the DEP force, the fewer cycles that were required to reach the maximum enhancement in capture rate. Stronger DEP forces therefore coat the surface of the pipette with DNA at a much faster rate than smaller DEP forcing conditions.

Supplementary References

1. Ling DY, Ling XS. On the distribution of DNA translocation times in solid-state nanopores: an analysis using Schrödinger's first-passage-time theory. *J. Phys. Condens. Matter* **25**, 375102 (2013).

2. Morgan H, Green NG. *AC Electrokinetics: Colloids and Nanoparticles*. Research Studies Press: Philadelphia, PA, USA, (2003).

3. van Dorp S, Keyser UF, Dekker NH, Dekker C, Lemay SG. Origin of the electrophoretic force on DNA in solid-state nanopores. *Nat. Physics* **5**, 347-351 (2009).

4. Bakewell DJ, Ermolina I, Morgan H, Milner J, Feldman Y. Dielectric relaxation measurements of 12 kbp plasmid DNA. *Biochim. Biophys. Acta* **1493**, 151-158 (2000).

5. Bakewell DJ, Vergara-Irigaray N, Holmes D. Dielectrophoresis of biomolecules. *Nanotechnol. Nanomed.* **1**, 1003 (2013).

6. Pohl HA. *Dielectrophoresis : the Behavior of Neutral Matter in Nonuniform Electric Fields*. Cambridge University Press (1978).

7. Nicoli F, Verschueren D, Klein M, Dekker C, Jonsson MP. DNA translocations through solid-state plasmonic nanopores. *Nano Lett.* **14**, 6917-6925 (2014).

8. Keyser UF, Krapf D, Koeleman BN, Smeets RM, Dekker NH, Dekker C. Nanopore tomography of a laser focus. *Nano Lett.* **5**, 2253-2256 (2005).

9. Chen P, Gu J, Brandin E, Kim Y-R, Wang Q, Branton D. Probing single DNA molecule transport using fabricated nanopores. *Nano Lett.* **4**, 2293-2298 (2004).

10. Wanunu M, Morrison W, Rabin Y, Grosberg AY, Meller A. Electrostatic focusing of unlabelled DNA into nanoscale pores using a salt gradient. *Nat. Nanotechnol.* **5**, 160-165 (2010).

11. Smeets RM, Keyser UF, Dekker NH, Dekker C. Noise in solid-state nanopores. *Proc. Natl. Acad. Sci. USA* **105**, 417-421 (2008).

12. Laohakunakorn N, Thacker VV, Muthukumar M, Keyser UF. Electroosmotic flow reversal outside glass nanopores. *Nano Lett.* **15**, 695–702 (2014).

13. Smeets RM, Keyser UF, Krapf D, Wu M-Y, Dekker NH, Dekker C. Salt dependence of ion transport and DNA translocation through solid-state nanopores. *Nano Lett.* **6**, 89-95 (2006).

14. Chang H, Kosari F, Andreadakis G, Alam M, Vasmatzis G, Bashir R. DNA-mediated fluctuations in ionic current through silicon oxide nanopore channels. *Nano Lett.* **4**, 1551-1556 (2004).

15. Gershow M, Golovchenko J. Recapturing and trapping single molecules with a solid-state nanopore. *Nat. Nanotechnol.* **2**, 775-779 (2007).

16. Panja D, Barkema GT, Kolomeisky AB. Through the eye of the needle: recent advances in understanding biopolymer translocation. *J. Phys. Condens. Matter.* **25**, 413101 (2013).
